# RNA-Seq analysis of duck embryo fibroblast cells gene expression during duck Tembusu virus infection

**DOI:** 10.1186/s13567-022-01051-y

**Published:** 2022-05-18

**Authors:** Yuhong Pan, Xuedong Wu, Wenjun Cai, Anchun Cheng, Mingshu Wang, Shun Chen, Juan Huang, Qiao Yang, Ying Wu, Di Sun, Sai Mao, Dekang Zhu, Mafeng Liu, Xinxin Zhao, Shaqiu Zhang, Qun Gao, Xumin Ou, Bin Tian, Zhongqiong Yin, Renyong Jia

**Affiliations:** 1 Research Center of Avian Disease, College of Veterinary Medicine, Sichuan Agricultural University, Chengdu, 611130 Sichuan China; 2 Institute of Preventive Veterinary Medicine, Sichuan Agricultural University, Chengdu, 611130 Sichuan China; 3 Key Laboratory of Animal Disease and Human Health of Sichuan Province, Chengdu, 611130 Sichuan China

**Keywords:** RNA-seq, duck Tembusu virus, duck embryo fibroblast cells, virus infection, immune responses

## Abstract

Duck Tembusu virus (DTMUV), a member of the family *Flaviviridae* and an economically important pathogen with a broad host range, leads to markedly decreased egg production. However, the molecular mechanism underlying the host-DTMUV interaction remains unclear. Here, we performed high-throughput RNA sequencing (RNA-Seq) to study the dynamic changes in host gene expression at 12, 24, 36, 48 and 60 h post-infection (hpi) in duck embryo fibroblasts (DEF) infected with DTMUV. A total of 3129 differentially expressed genes (DEG) were identified after DTMUV infection. Gene Ontology (GO) category and Kyoto Encyclopedia of Genes and Genomes (KEGG) pathway enrichment analysis revealed that these DEG were associated with multiple biological functions, including signal transduction, host immunity, virus infection, cell apoptosis, cell proliferation, and pathogenicity-related and metabolic process signaling pathways. This study analyzed viral infection and host immunity induced by DTMUV infection from a novel perspective, and the results provide valuable information regarding the mechanisms underlying host-DTMUV interactions, which will prove useful for the future development of antiviral drugs or vaccines for poultry, thus benefiting the entire poultry industry.

**Supplementary Information:**

The online version contains supplementary material available at 10.1186/s13567-022-01051-y.

## Introduction

Duck Tembusu virus (DTMUV) is an arbovirus of the genus *Flavivirus*, family *Flaviviridae*. DTMUV is an enveloped virus with a single-stranded, positive-polarity RNA genome that is approximately 11 kb in length [1]. Beginning in April 2010, an outbreak of DTMUV occurred in major duck farming regions in China [2]. Since then, DTMUV has also caused serious damage to the duck industry in Southeast Asian countries Thailand and Malaysia, as well as in China [3–5]. The slow growth of poultry, the sharp decline in egg production, and even the suspension of production have caused major economic losses [6]. Several studies have revealed that DTMUV has a wide host range, infecting ducks, chickens, geese, pigeons and house sparrows [7, 8]. Moreover, more than 70% of duck industry workers were reported to have Abs against DTMUV in the serum samples tested, and ~50% of oral swab samples were found to be positive for DTMUV RNA [9]. These studies clearly prove that DTMUV is likely to spread from ducks to other non-avian hosts and even humans. Therefore, it is necessary to explore the pathogenic mechanisms of DTMUV to allow the design of better disease control strategies.

China raises the largest number of ducks among all countries worldwide. Ducks are a natural host of DTMUV, as DTMUV and DTMUV antibodies have been found repeatedly in various domesticated ducks, and global serological evidence of DTMUV infection has been reported in a variety of wild waterfowl species [7, 10]. Duck embryo fibroblasts are the primary target cells of DTMUV and play a vital role in in vitro studies of host-DTMUV interactions [11, 12]. However, few studies have used DEF to investigate differential gene expression in response to DTMUV infection, which may provide important information about host-DTMUV interactions at the transcriptional level and in terms of biological processes, molecular functions, and cellular components. Very recently, next-generation sequencing techniques such as RNA-Seq, which is a powerful approach for transcriptome profiling, have revealed dynamic changes in host gene expression during pathogen infections and have been employed to study various viral infections and diseases [13–15].

The aim of the current study was to use the RNA-Seq method to conduct a comparative transcriptome analysis of DEF in response to DTMUV infection at different time points (12, 24, 36, 48 and 60 hpi) and to analyze the dynamics of host gene expression during viral infection. The results may provide novel information that will increase our understanding of the pathogenesis of DTMUV and the mechanisms underlying virus-host interactions.

### Materials and methods

#### Cell culture and virus infection

Duck embryo fibroblasts were obtained from 10-day-old duck embryos according to the manufacturer’s instructions [16]. In this study, DEF were cultured in plates containing Dulbecco modified Eagle medium (DMEM, Gibco, MD, USA) supplemented with 10% newborn bovine serum (NBS, Gibco) at 37 ℃ in a 5% CO_2_ atmosphere. When DEF reached ~90% confluence, they were mock-infected or infected with DTMUV CQW1 strain (GenBank: KM233707.1) at a multiplicity of infection (MOI) of 1 [17, 18]. After the virus was adsorbed in a 37 °C, 5% CO_2_ incubator for 1 h, the inoculum was replaced with maintenance medium (DMEM containing 2% NBS), and the cell samples were collected at 12, 24, 36, 48 and 60 hpi, respectively. All duck embryos used in this study were selected in accordance with the guidelines provided by the Chinese Council on Animal Care.

#### RNA isolation, cDNA library construction and RNA-Seq

Cellular and viral RNA were extracted with Trizol reagent (Invitrogen, Life Technologies, CA, USA) according to the manufacturer’s protocol and subsequently treated with DNase I (Invitrogen, Life Technologies). RNA purity, concentration, and integrity were assessed using a Nano drop ND-1000 spectrophotometer (Nano drop Technologies, CA, USA), a Qubit 2.0 fluorometer (Invitrogen, Life Technologies), and an Agilent 2100 bioanalyzer (Agilent, CA, USA), respectively. A cDNA library for each sample was constructed with an RNA Library Prep Kit (NEB, USA) according to the manufacturer’s instructions. Next, 3 μg of RNA was used to enrich poly (A) mRNA, which was fragmented into short pieces by oligo (dT) magnetic beads (Invitrogen, Life Technologies). Cleaved short RNA fragments were used for first-strand cDNA synthesis with reverse transcriptase and a random hexamer primer. Second-strand cDNA fragments were then synthesized using DNA polymerase I, RNase H, and dNTP. The cDNA fragments were ligated to sequencing adapters and amplified by PCR to obtain the final paired-end library. Library sequencing was performed on the Illumina sequencing platform (HiSeq 2500) to generate 150 bp paired-end reads.

#### Transcriptome data analysis

To analyze the RNA-Seq data, low-quality sequences were removed from the raw sequencing reads, and the adaptor sequences were trimmed using the Sequence CLEAN program. The remaining reads were called “clean reads” and stored in FASTQ format. The assembled unigenes were then mapped to mallard (*Anas platyrhynchos*) genome (GenBank: NM_001005484.1) using TopHat2 software [19]. After that, the transcripts were assembled with cufflinks [20]. Differences in gene expression levels were standardized by the reads per kilobase of unigene per million mapped reads (FPKM) method. The NOISeq [21] method was used to screen the differentially expressed genes (DEG) according to default criteria consisting in a |log_2_ Fold Change|> 1 and a *p* value < 0.05. Pathway annotation and enrichment analyses were performed using the Gene Ontology (GO) and Kyoto Encyclopedia of Genes and Genomes (KEGG) pathway databases. All presented data represent average changes in gene expression for 3 independent replicates.

### Results

#### DTMUV replication in DEF

**Figure 1:**
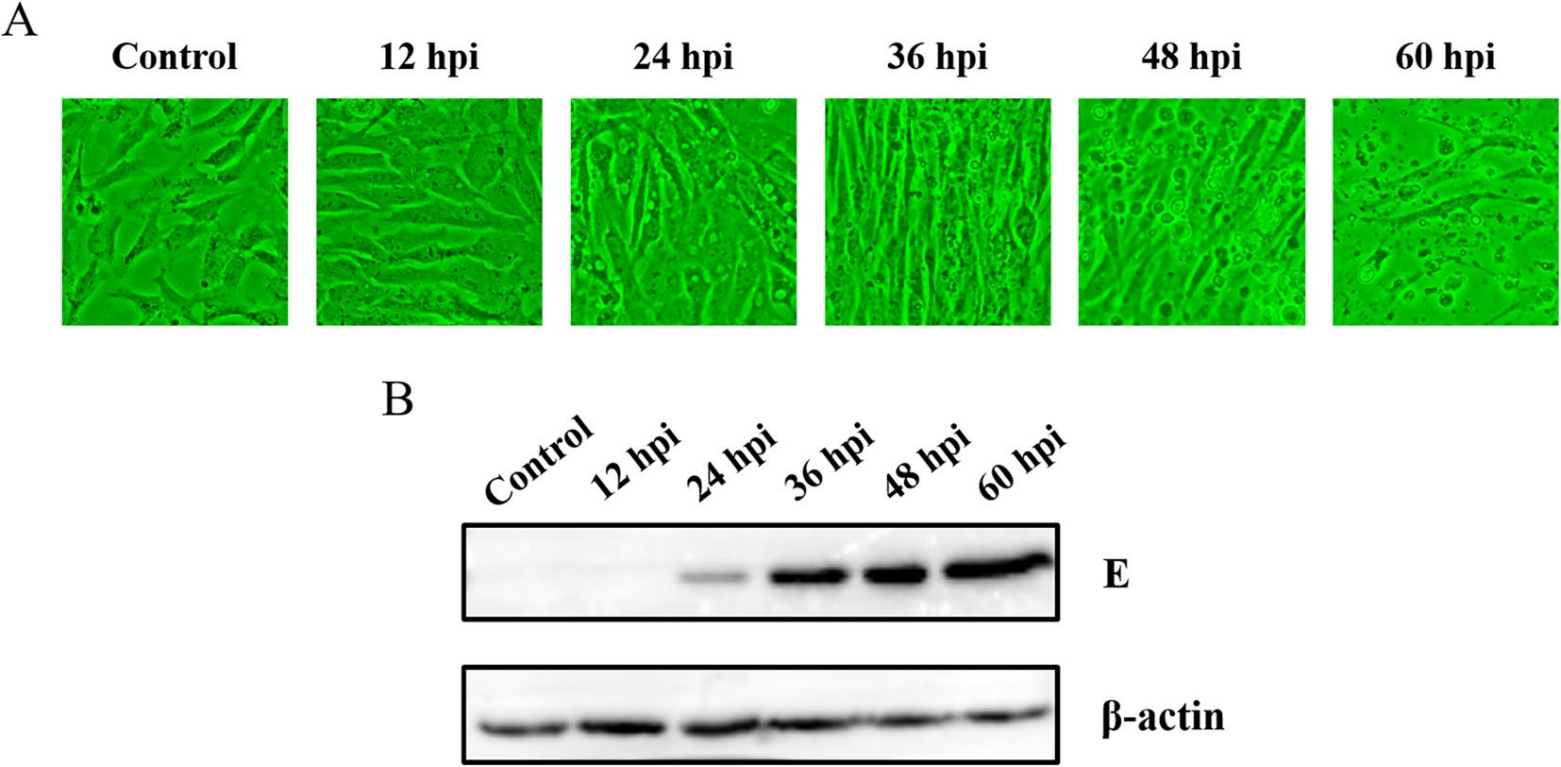
**DTMUV infection in DEF. A** The cytopathic effects (CPE) of DEF after DTMUV infection at 12, 24, 36, 48 and 60 hpi, and mock infected cells as the control.** B** Western blot analysis was used to detect the expression of E protein in DTMUV-infected DEF and control cells. The protein samples were separated by SDS-PAGE and transferred to PVDF membranes. The blots were incubated with a mouse monoclonal antibody against DTMUV envelop protein E. The expression of β-actin was used as an internal control.

DTMUV infection in DEF was confirmed by cytopathic effects (CPE) and Western blot. First, the morphological changes in DTMUV-infected DEF were determined by microscopic observations 12, 24, 36, 48, and 60 hpi. As shown in Figure 1A, we observed that DTMUV caused minimal CPE in DEF cells at 24 hpi. At 36, 48, and 60 hpi, compared with the morphology of the control cells, obvious cellular fragmentation and increased granularity were observed in the DTMUV-infected DEF. As shown in Figure 1B, the viral E protein expression was detected at 24 hpi, and greater expression was observed at 36, 48 and 60 hpi by Western blot.

#### RNA sequencing and read assembly

To study DTMUV-DEF interactions, sequencing libraries were prepared in triplicate for 5 different time points after DEF were infected with DTMUV (12, 24, 36, 48 and 60 hpi) and were analyzed in comparison with uninfected cells at the corresponding time. In these experiments, an average of 47939795 clean sequencing reads were generated. After discarding ribosomal RNA and low-quality reads, 30 high-quality samples were further mapped onto the *Anas platyrhynchos* genome (GenBank accession: NM_001005484.1) using the TopHat2 software (Table 1). The average ratio of high-quality reads in comparison with the reference genome was 85.26%. Qualifying sample data were evaluated using multiple metrics (Table 1). The raw sequencing data have been deposited online in SRA at the NCBI under accession number (SRS6277135).

**Table 1:** **Statistics of the RNA-seq datasets**.

Sample	Clean reads	Total mapped reads	Multiple mapped reads	Unique mapped reads	Q30	GC
12 hpi-C1	48 274 628	41 573 336 (86.12%)	6 378 708 (13.21%)	35 194 628 (72.91%)	94.70%	48.42%
12 hpi-C2	47 830 078	41 308 712 (86.37%)	5 737 883 (12.00%)	35 570 829 (74.37%)	94.71%	48.26%
12 hpi-C3	48 078 544	41 460 249 (86.23%)	5 979 168 (12.44%)	35 481 081 (73.80%)	94.62%	48.32%
12 hpi-V1	47 797 726	41 749 761 (87.35%)	5 158 422 (10.79%)	36 591 339 (76.55%)	94.56%	46.81%
12 hpi-V2	48 662 096	42 265 846 (86.86%)	6 254 508 (12.85%)	36 011 338 (74.00%)	94.90%	47.41%
12 hpi-V3	48 357 954	41 691 562 (86.21%)	5 645 201 (11.67%)	36 046 361 (74.54%)	94.51%	47.84%
24 hpi-C1	47 831 630	41 436 320 (86.63%)	7 187 040 (15.03%)	34 249 280 (71.60%)	94.44%	48.00%
24 hpi-C2	47 774 482	41 046 949 (85.92%)	6 987 440 (14.63%)	34 059 509 (71.29%)	94.21%	48.66%
24 hpi-C3	47 838 498	41 433 460 (86.61%)	7 225 949 (15.10%)	34 207 511 (71.51%)	94.29%	48.05%
24 hpi-V1	47 928 196	41 001 644 (85.55%)	5 782 993 (12.07%)	35 218 651 (73.48%)	94.62%	48.16%
24 hpi-V2	48 096 832	41 158 867 (85.58%)	5 466 059 (11.36%)	35 692 808 (74.21%)	94.56%	48.16%
24 hpi-V3	47 598 466	41 103 273 (86.35%)	5 592 406 (11.75%)	35 510 867 (74.61%)	94.36%	47.56%
36 hpi-C1	47 926 732	40 822 547 (85.18%)	6 487 685 (13.54%)	34 334 862 (71.64%)	93.97%	48.75%
36 hpi-C2	47 927 484	40 952 014 (85.45%)	6 565 301 (13.70%)	34 386 713 (71.75%)	93.97%	48.72%
36 hpi-C3	47 839 192	41 096 981 (85.91%)	6 323 919 (13.22%)	34 773 062 (72.69%)	94.25%	48.38%
36 hpi-V1	47 887 276	40 061 084 (83.66%)	5 321 156 (11.11%)	34 739 928 (72.55%)	94.23%	48.57%
36 hpi-V2	48 030 780	40 078 123 (83.44%)	4 911 209 (10.23%)	35 166 914 (73.22%)	94.52%	48.66%
36 hpi-V3	48 384 374	40 561 234 (83.83%)	5 180 225 (10.71%)	35 381 009 (73.12%)	94.54%	48.31%
48 hpi-C1	47 562 858	40 922 500 (86.04%)	8 274 378 (17.40%)	32 648 122 (68.64%)	94.36%	48.08%
48 hpi-C2	47 742 022	41 125 020 (86.14%)	7 600 905 (15.92%)	33 524 115 (70.22%)	93.74%	48.20%
48 hpi-C3	47 743 490	40 844 737 (85.55%)	7 514 714 (15.74%)	33 330 023 (69.81%)	94.20%	48.30%
48 hpi-V1	47 739 426	39 938 122 (83.66%)	5 678 207 (11.89%)	34 259 915 (71.76%)	94.03%	48.35%
48 hpi-V2	47 930 590	40 096 360 (83.66%)	5 657 021 (11.80%)	34 439 339 (71.85%)	93.75%	48.14%
48 hpi-V3	47 672 068	39 927 761 (83.76%)	5 902 585 (12.38%)	34 025 176 (71.37%)	93.89%	48.17%
60 hpi-C1	47 730 090	40 835 692 (85.56%)	7 252 298 (15.19%)	33 583 394 (70.36%)	94.06%	48.24%
60 hpi-C2	47 944 660	40 693 994 (84.88%)	7 032 556 (14.67%)	33 661 438 (70.21%)	94.08%	48.44%
60 hpi-C3	48 372 046	41 250 387 (85.28%)	7 097 697 (14.67%)	34 152 690 (70.60%)	94.18%	48.35%
60 hpi-V1	47 806 440	39 672 477 (82.99%)	4 589 271 (9.60%)	35 083 206 (73.39%)	93.90%	48.04%
60 hpi-V2	47 714 394	39 810 899 (83.44%)	4 873 440 (10.21%)	34 937 459 (73.22%)	94.11%	47.74%
60 hpi-V3	48 170 796	40 314 245 (83.69%)	4 852 943 (10.07%)	35 461 302 (73.62%)	93.65%	47.88%

Clean Reads: The number of sequencing sequences after filtering.

Total Mapped Reads: The number of sequencing sequences that can be mapped to the genome.

Multiple Mapped Reads: The number of sequencing sequences that have multiple alignment positions on the reference sequence.

Unique Mapped Reads: The number of sequencing sequences that have unique alignment positions on the reference sequence.

#### Global changes of gene expression after DTMUV infection

**Figure 2:**
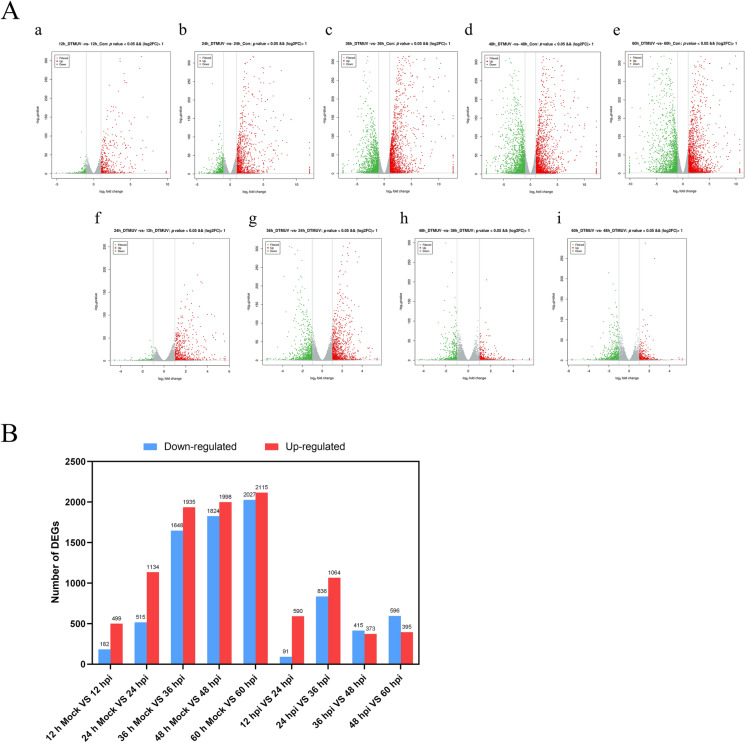
**Changes of the gene expression in DEF at different times after DTMUV infection.** **A** Volcano chart of differentially expressed genes (DEG). The x-axis shows the log_2_ (fold change) and y-axis shows the -log_10_ (*p*-value). Red dots represent the upregulated DEG, green dots represent the downregulated DEG and gray dots are the no significant difference genes (a. 12 h Mock VS 12 hpi; b. 24 h Mock VS 24 hpi; c. 36 h Mock VS 36 hpi; d. 48 h Mock VS 48 hpi; e. 60 h Mock VS 60 hpi; f. 12 hpi VS 24 hpi; g. 24 hpi VS 36 hpi; h. 36 hpi VS 48 hpi; i. 48 hpi VS 60 hpi). The vertical line in the figure is the two-fold expression difference threshold; the horizontal line is the *p*-value = 0.05 threshold. **B** The up-down statistical chart of the DEG.

We use the volcano plot (Figure 2A) and histogram (Figure 2B) to evaluate the changes in the mRNA expression profile of DEF after DTMUV infection for 12, 24, 36, 48 and 60 hpi (12 h Mock VS 12 hpi, 24 h Mock VS 24 hpi, 36 h Mock VS 36 hpi, 48 h Mock VS 48 hpi, 60 h Mock VS 60 hpi, 12 hpi VS 24 hpi, 24 hpi VS 36 hpi, 36 hpi VS 48 hpi, 48 hpi VS 60 hpi). At 12 h after DTMUV infection of DEF, there were 499 up-regulated genes and 182 down-regulated genes (Additional file 1); at 24 hpi, there were 1134 up-regulated genes and 515 down-regulated genes (Additional file 2); at 36 hpi, there were 1935 up-regulated genes and 1648 down-regulated genes (Additional file 3); at 48 hpi, there were 1998 up-regulated genes and 1824 down-regulated genes (Additional file 4); at 60 hpi, there were 2115 up-regulated genes and 2027 down-regulated genes (Additional file 5). In addition, a comparison of 24 hpi with 12 hpi shows that 590 genes were up-regulated and 91 genes were down-regulated. A comparison of 36 hpi with 24 hpi shows that there were 1064 up-regulated genes and 836 down-regulated genes. A comparison of 48 hpi with 36 hpi show that 373 genes were up-regulated and 415 genes were down-regulated 48 h after DTMUV infection and a comparison of 60 hpi with 48 hpi shows that 395 genes were up-regulated and 596 genes were down-regulated. We can see that after 36 h of DTMUV infection, the number of DEG increased significantly.

#### Significantly differentially expressed transcripts and clustering

**Figure 3:**
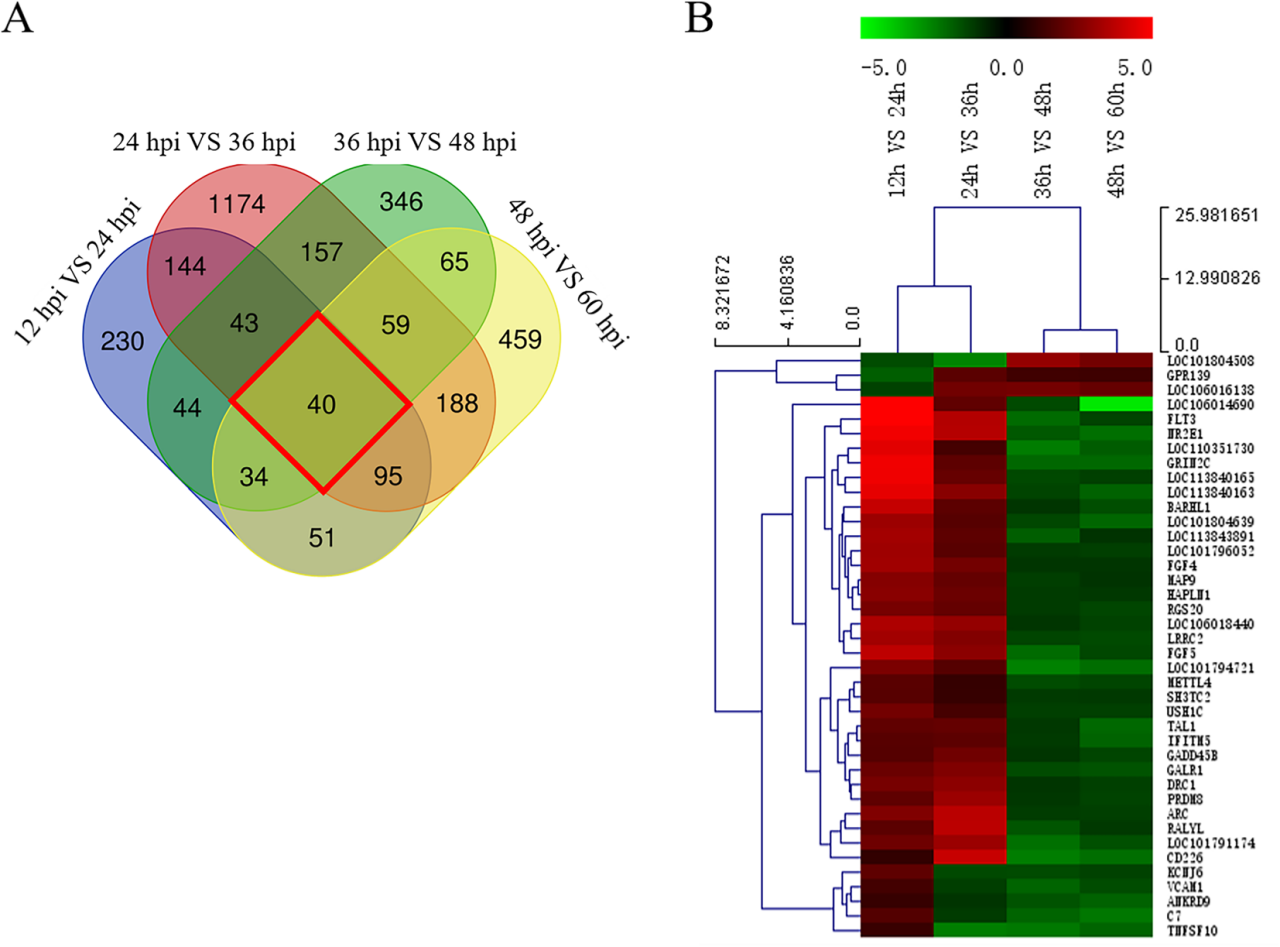
**Transcriptome data profile generated by Illumina HiSeqTM 2500 platform and differential expression analysis.** **A** Venn diagram showing unique and co-differentially expressed genes in response to DTMUV infection at 12 and 24 hpi, 24 and 36 hpi, 36 and 48 hpi, and 48 and 60 hpi; **B** Hierarchical clustering analysis of co-differentially expressed genes at 12 and 24 hpi, 24 and 36 hpi, 36 and 48 hpi, and 48 and 60 hpi. The color in the heat map represents gene expression changes. Red indicates upregulation of gene expression, green indicates downregulation of expression.

In order to more intuitively reflect the changes in DEG that accompany the process of viral infection, we established a Venn diagram to delve deeper into genes that are unique or shared during DTMUV infection (Figure 3A). The Venn diagram analysis revealed that 40 DEG were observed at all tested time points and that 230, 1174, 346 and 459 DEG were unique when comparing 12 and 24 hpi, 24 and 36 hpi, 36 and 48 hpi, and 48 and 60 hpi, respectively. In Figure 3A, genes that were differentially expressed at all time points were called continuous upregulated and downregulated genes and are marked with red circles; the DEG in this group undoubtedly play an important role during DTMUV infection. Hierarchical clustering was performed to compare a total of 40 DEG that were differentially expressed in DEF during different phases of DTMUV infection. Gradual changes in gene expression were observed between 12 and 24 hpi, 24 and 36 hpi, 36 and 48 hpi, and 48 and 60 hpi, and the left side of the hierarchical clustering gene tree allowed DEG to be further classified (Figure 3B).

#### GO analyses of DEG

**Figure 4:**
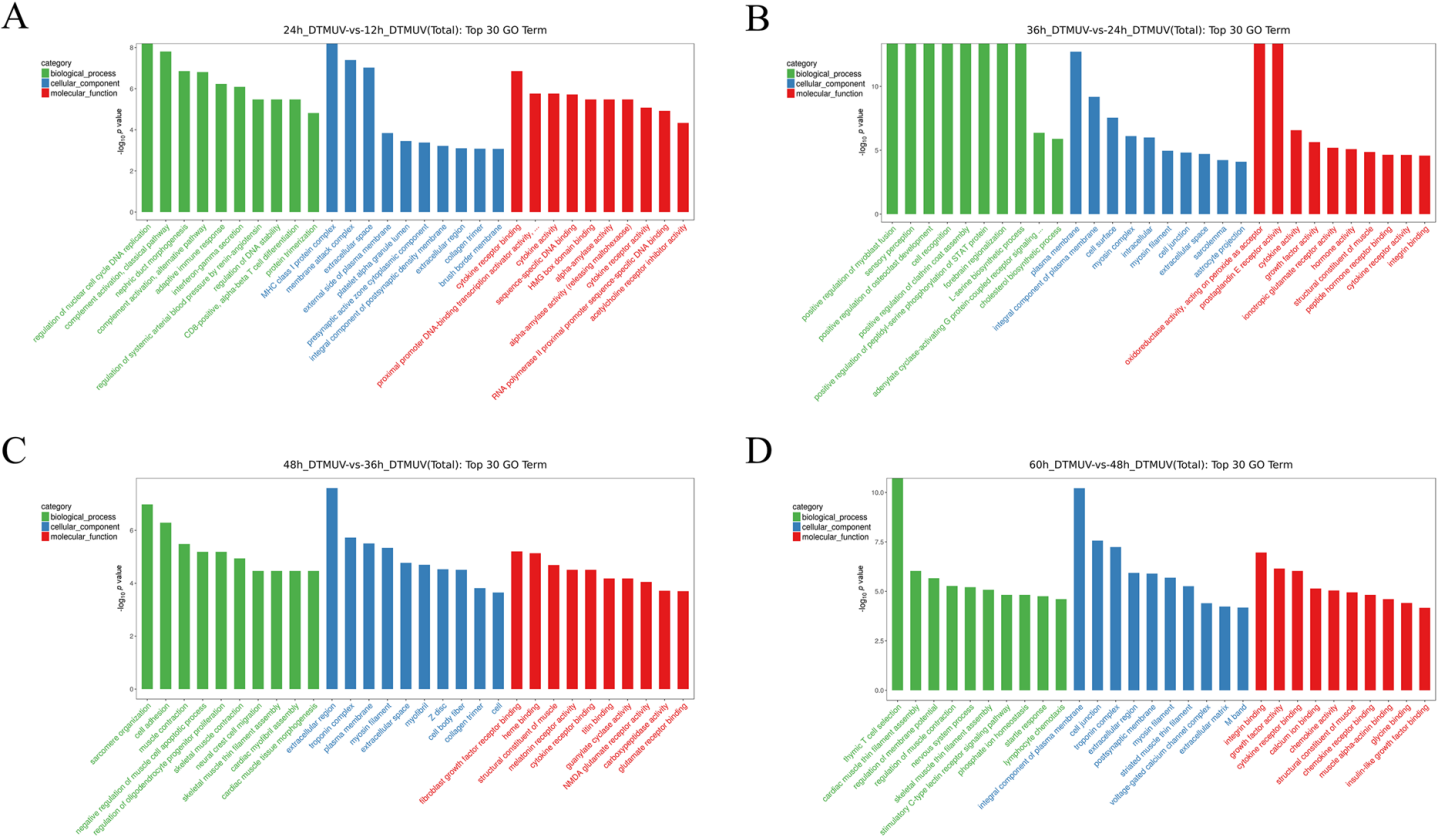
**Top 30 Gene ontology (GO) terms of DEG expressed in DTMUV-infected DEF.** **A** GO annotation of DEG expressed at 12 and 24 hpi; **B** GO annotation of DEG expressed at 24 and 36 hpi; **C** GO annotation of DEG expressed at 36 and 48 hpi; **D** GO annotation of DEG expressed at 48 and 60 hpi. GO terms were classified into 3 categories, including cellular component (CC), molecular function (MF), and biological process (BP). The top 30 GO terms were selected according to *p*-value < 0.05.

A total of 3129 DEG were analyzed using the GO database at all the time points. The GO functional analysis resulted in 2797 GO terms being assigned to 12 and 24 hpi, including 320 GO terms in the cellular component (CC) category, 581 GO terms in the molecular functions (MF) category, and 1896 terms in the biological process (BP) category. A total of 5818 GO terms were assigned to 24 and 36 hpi, including 620 GO terms in the CC category, 1221 GO terms in the MF category, and 3977 GO terms in the BP category. A total of 3030 GO terms were assigned to 36 and 48 hpi, including 392 GO terms in the CC category, 696 GO terms in the MF category, and 1942 GO terms in the BP category. A total of 3506 GO terms were assigned to 48 and 60 hpi, including 418 GO terms in the CC category, 748 GO terms in the MF category, and 2340 GO terms in the BP category. Next, to select the most useful genes for further investigation, we chose the top 30 enriched GO terms, listed in Figures 4A–D.

#### KEGG pathway analysis of DEG

**Figure 5:**
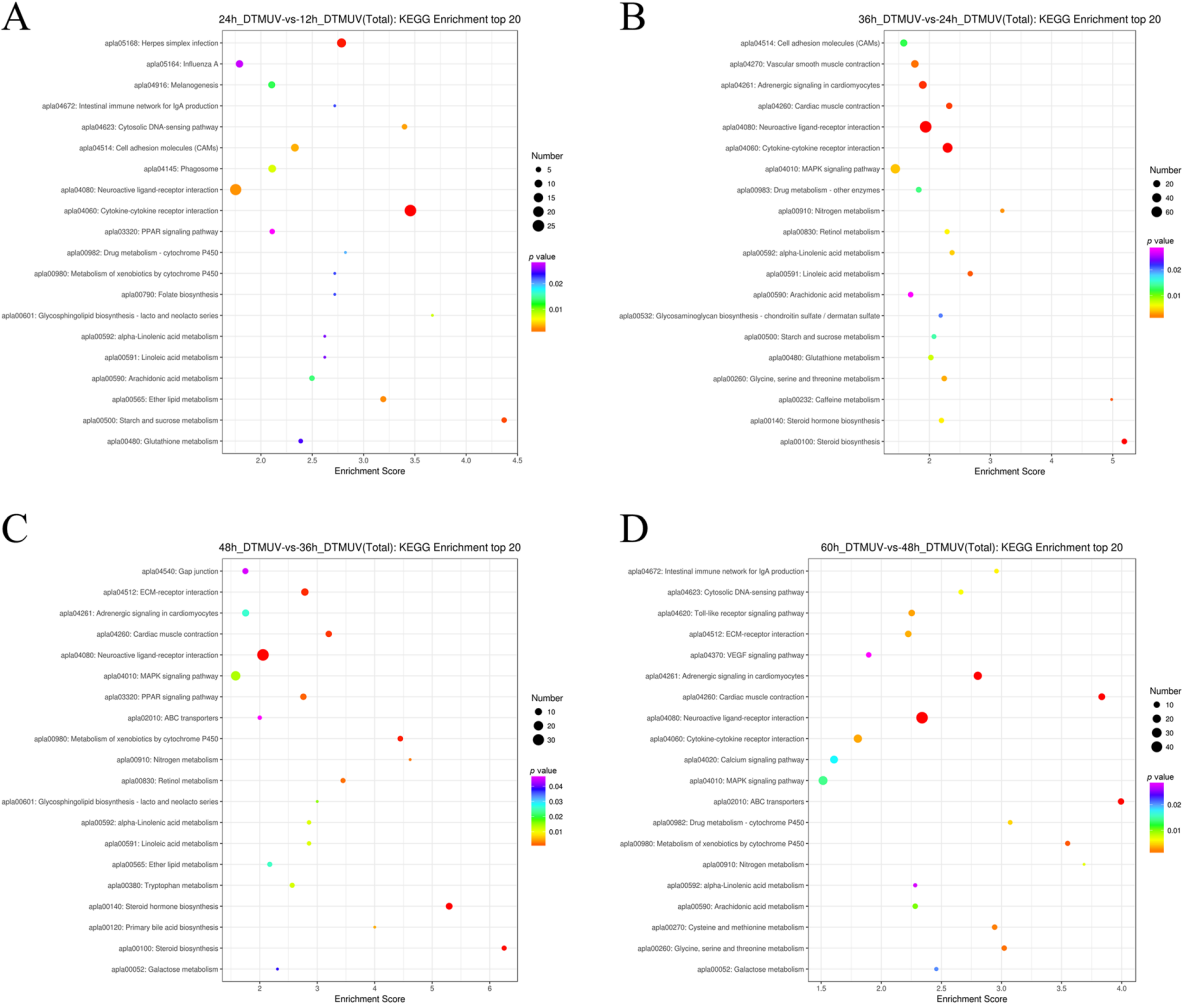
**Top 20 KEGG pathways in DTMUV-infected DEF.** **A** KEGG pathways of DEG expressed at 12 and 24 hpi; **B** KEGG pathways of DEG expressed at 24 and 36 hpi; **C** KEGG pathways of DEG expressed at 36 and 48 hpi; **D** KEGG pathways of DEG expressed at 48 and 60 hpi.

The KEGG database is used for the pathway-based classification of orthologous genes to provide useful information for predicting biological processes and phenotypic traits of genes. To determine the various biological processes involved in an DTMUV infection, 3129 DEG were mapped to referential canonical signaling pathways via KEGG database analysis. Of the differentially expressed DEF genes observed by comparing the 12 and 24 hpi, 188 DEG were mapped to 112 KEGG pathways. When comparing the 24 and 36 hpi, 554 DEG were mapped to 148 KEGG pathways. When comparing the 36 and 48 hpi, 230 DEG were mapped to 114 KEGG pathways. Finally, when comparing the 48 and 60 hpi, 288 DEG were mapped to 115 KEGG pathways. Next, we selected the top 20 most enriched KEGG pathways at all different time points according to a *p*-value < 0.05, as shown in Figures 5A–D. In addition, we screened out important DEG related to viral invasion (Additional file 6) and host cell defense pathways (Additional file 7).

## Discussion

Although many researchers have undertaken extensive efforts to understand flavivirus infection in human cells, little is known about DTMUV infection in avian-derived cells. As an economically important disease of the poultry industry, DTMUV has a broad host range and causes reduced egg production in ducks, leading to substantial economic losses [8]. However, little information has been reported on the molecular mechanism underlying the host-DTMUV interaction. In this study, we employed high-throughput RNA-Seq technology and explored important information about host-virus interaction at 5 characteristic stages (12, 24, 36, 48 and 24 hpi) of DTMUV-infected DEF.

**Figure 6:**
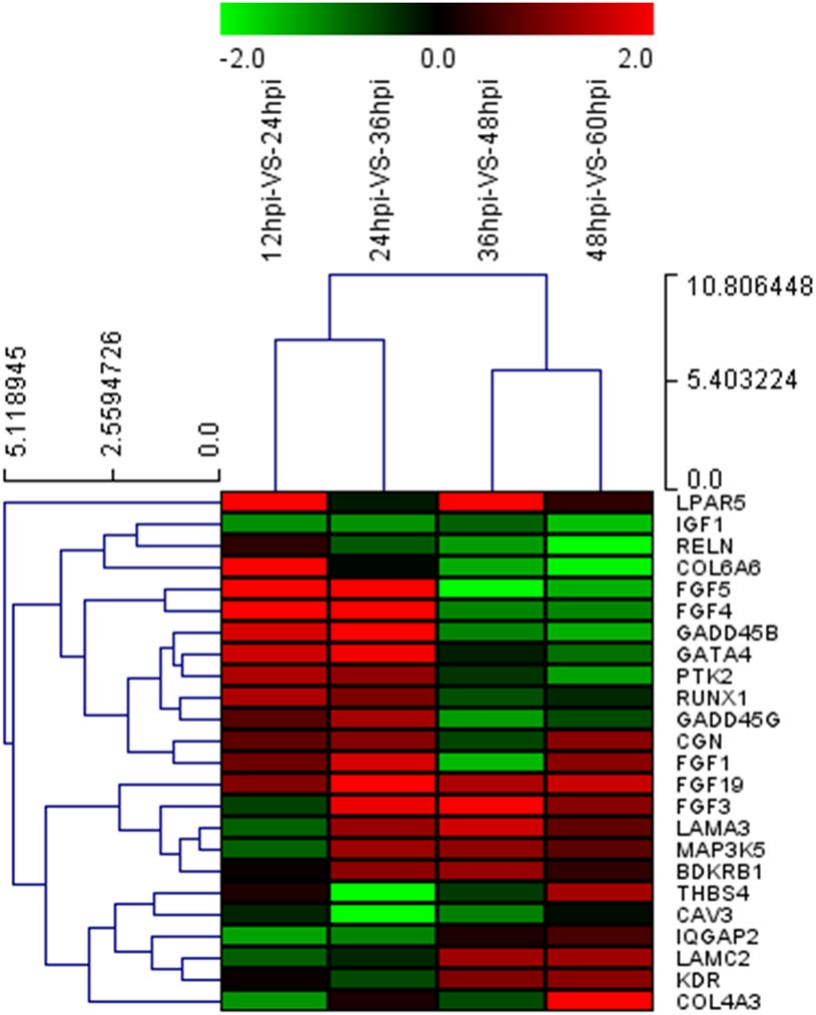
**Expression profiles (heat map) of virus infection genes at 12 and 24 hpi, 24 and 36 hpi, 36 and 48 hpi, 48 and 60 hpi.** The color in the heat map represents gene expression changes (red, high gene expression; green, low gene expression; black, no gene expression). DEG are labeled on the right of the heat-map, DEG clusters are labeled on the left of the heat-map.

**Figure 7:**
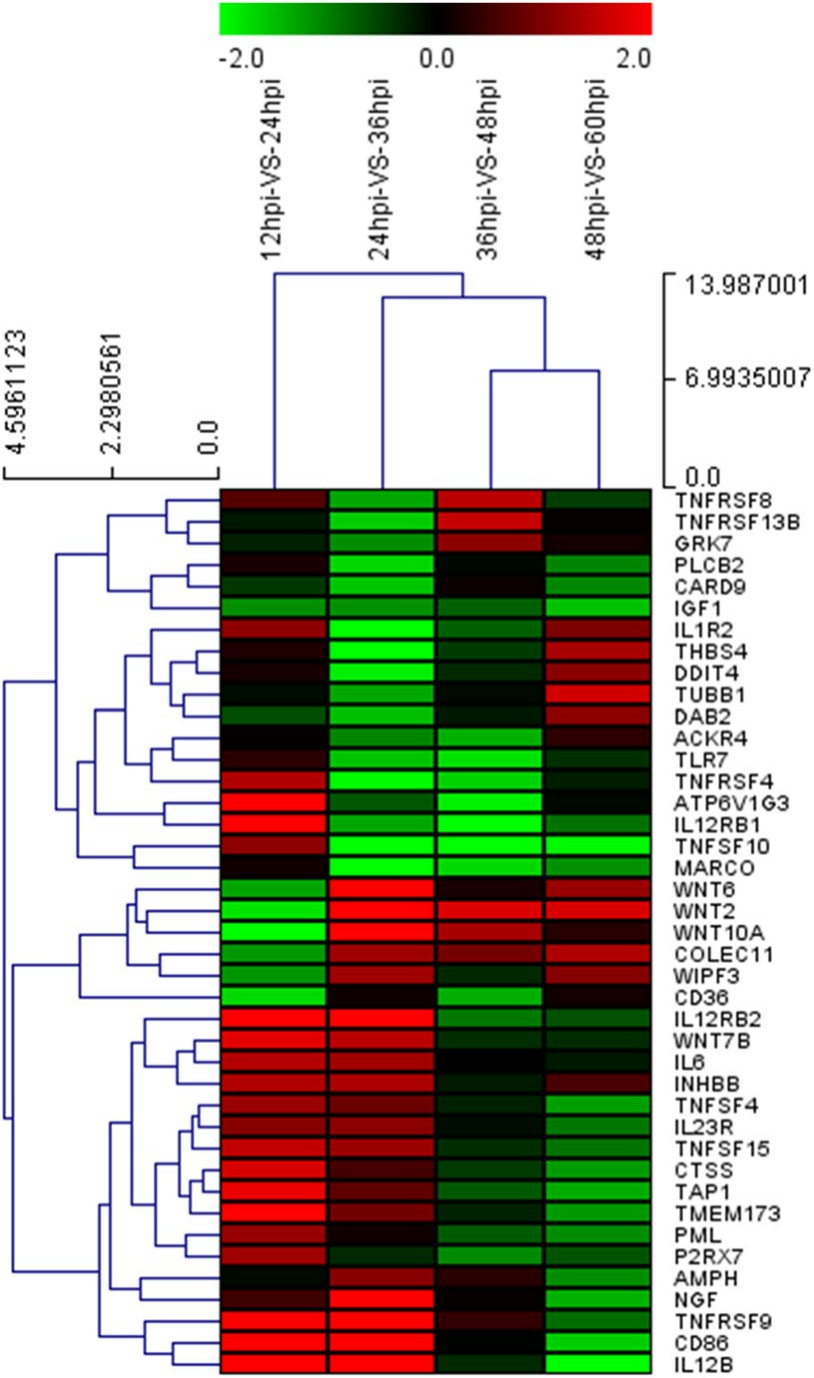
**Expression profiles (heat map) of host cells immune defense genes at 12 and 24 hpi, 24 and 36 hpi,** 36 and 48 hpi, 48 and 60 hpi. The color in the heat map represents gene expression changes (red, high gene expression; green, low gene expression; black, no gene expression). DEG are labeled on the right of the heat-map, DEG clusters are labeled on the left of the heat-map.

After DTMUV infection, the viruses first adhere to the cell surface and bind to specific receptors, a step that is considered a prelude to viral infection, and then enter the cell through endocytosis. Similarly, our study also detected the differential expression of CAV3, PML, AMPH, GRK7, WIPF3 and DAB2, which are associated with the endocytosis pathway induced by DTMUV (Figures 6 and 7). Then, we found that CGN, MAP3K5, GATA4 and RUNX1 related to tight junction were upregulated during DTMUV infection, indicating that these genes may play an important role in DTMUV entry.

The suppression of host cell growth is an important indicator of flavivirus infection [22–24]. In our study, we observed that the expression of cell cycle-associated DEG (GADD48B and GADD45G) was significantly downregulated at 48 and 60 hpi (Figure 6), indicating that the decreased expression of these genes may facilitate DTMUV replication. Most of the genes involved in the proinflammatory response were downregulated during DTMUV infection at 48 and 60 hpi (Figure 7), suggesting that flavivirus gene expression interfered with host cell antiviral defense mechanisms [25, 26]. Apoptosis is a critical defense mechanism in many organisms. Direct or indirect inhibition of apoptosis can facilitate viral infections [27, 28]. Moreover, some virus-induced apoptosis can enhance the spread of the virus, leading to tissue damage and disease. A previous morphological study revealed that DTMUV infection could cause apoptosis in DEF [8]. However, the molecular mechanism by which apoptosis is induced by DTMUV infection remains unclear. In this study, the IGF1, which regulates cell growth and proliferation, acting as a potent inhibitor of apoptosis [29], was clearly downregulated during DTMUV infection (Figure 6). Our results may be related to the facilitation of DTMUV infection. Future verification of the role of the IGF1 gene in regulating apoptosis in DTMUV-infected host cells is necessary.

To accomplish viral replication, assembly, and maturation, intracellular complexes of the virus utilize intracellular cytoskeletal components of the host to move from the periphery of the cell to sites of RNA synthesis [30]. Microtubules and actin are important components of the cellular cytoskeleton that facilitates flavivirus intracellular translocation to the nucleus [31–33]. In our study, we observed that DTMUV-infected DEF included a number of upregulated DEG that regulate the action cytoskeleton signaling pathway, such as FGF (fibroblast growth factor) family members (FGF1/3/4/5/19) and scaffold protein IQGAP2, which play a role in stabilizing microtubules [34–36] (Figure 6). Then, we found that tight junction (CGN, MAP3K5, GATA4 and RUNX1) and focal adhesion (KDR, LAMA3, LAMC2, PTK2, COL6A6 and COL4A3) play important roles in DTMUV replication, which is similar to the reports of flavivirus [37, 38].

Upon DTMUV infection, host defense against viral infections occurs via several different mechanisms, primarily involving the innate immune response at early time points and shifting to adaptive immune responses at later time points. The innate immune response is activated by pattern recognition receptors that recognize conserved microbial molecules known as pathogen-associated molecular patterns, constituting an early line of defense [39]. In our study, as a result of DTMUV invasion, the Nod-like receptor pathway (P2RX7, TMEM173 and IL6), the Toll-like receptor pathway (TLR7, CD86, IL6 and IL12B), and the RIG-I-like receptor pathway (TMEM173 and IL12B) were activated (Figure 7). In a recent study, Han et al. [40] found that the host innate immune system was also activated after DTMUV infection of DF-1 cells, including TLR pathway, RLR receptor pathway, NLR pathway and JAK-STAT pathway. Also, they found that numerous critical molecules in the TLR, RLR and NLR pathways were upregulated, such as TLR3, IRF7, STAT1, IFN-α and IFN-β. The ubiquitin—proteasome system (UPS) is the major degradation system in the host cells, also playing an important role in the different stages of the virus life cycle, including viral adsorption, viral penetration and uncoating, gene transcription, protein synthesis, assembly, and viral progeny release. Interestingly, Han et al. found that some UPS-related DEG (TRIM36, DTX3L, MYLIP, RNF185, TRIM25, and RNF19β) were significantly upregulated during DTMUV infection by RNA-seq technology, which may be related to their previous finding that UPS is involved in DTMUV replication [41].

Pathogen-associated molecular patterns are recognized by pattern recognition receptors, triggering a downstream signaling cascade and a proinflammatory response that includes cytokines, chemokines, and tumor necrosis factor [39, 42]. Our results indicate that the expression of IL6, IL12B, IL1R2, IL23R, IL12RB1 and IL12RB2 increased after DTMUV infection (Figure 7). IL6 and IL12B are 2 important inflammatory factors that are upregulated in DTMUV [43, 44]. However, dysregulated expression of IL6 or IL12B can cause several inflammatory diseases and death [45]. Additionally, tumor necrosis factor alpha (TNF-α) is a pleiotropic cytokine that produces a wide range of stimuli. TNFSF4/5/10 and TNFRSF4/8/9/13B were influenced during DTMUV infection and all these factors function as important proinflammatory genes and play vital roles in the inflammatory response [46, 47]. These results indicate that these important proinflammatory responses may activate the innate immune response and promote viral clearance. Further studies examining the mechanism underlying cytokine regulation in DTMUV-infected cells are necessary.

It is well accepted that complete clearance of intracellular viruses requires the destruction of infected cells by the adaptive immune system [48]. To the best of our knowledge, the phagosome plays an important role in balancing microbicidal and proteolytic degradation functions with the generation of antigenic peptides for presentation by MHCI and MHCII molecules to CD8 and CD4 T cells [49]. We found that the genes (ATP6V1G3, MARCO, TAP1, THBS4 and TUBB1) involved in the formation of phagosomes are up-regulated to varying degrees after DTMUV infection. MHCII molecules are transported to the plasma membrane and present viral peptides to CD4^+^ cells [50], and CD36 is required for proper MHCII antigen pentation [51, 52]. Our results indicate that CD36 and CTSS (the basic components of MHCII) were upregulated in DTMUV-infected cells, indicating that DTMUV has the potential to be presented to CD4 cells and evoke a host adaptive immune response as a defense against DTMUV infection. In addition, COLEC11, which is involved in the activation of the complement system, was also significantly up-regulated. Therefore, based on our study results, we infer that activation of the complement system and antigen presentation by DTMUV infection in DEF might serve as a link between the innate and adaptive immune responses to facilitate an integrated host response [53]. In addition, activation of the complement system and antigen presentation can help to increase the level of specific immune responses, providing new ideas for the design and development of new vaccines or vaccine adjuvants in the future.

This RNA-Seq study offers new insights and provides potential research targets for a better understanding of the DEF response to DTMUV infection. Our data highlight how viral infection relates to host defensive responses during virus infection. In a broader sense, the basic data obtained in this study are a valuable resource that provide a preliminary but comprehensive understanding of the complexity of the molecular mechanisms of the host-DTMUV interaction and provide new ideas for vaccine development.

## Supplementary Information

**Additional file 1: Information of differentially expressed genes at 12 hpi after DTMUV infection.**

**Additional file 2: Information of differentially expressed genes at 24 hpi after DTMUV infection.**

**Additional file 3: Information of differentially expressed genes at 36 hpi after DTMUV infection.**

**Additional file 4: Information of differentially expressed genes at 48 hpi after DTMUV infection.**

**Additional file 5: Information of differentially expressed genes at 60 hpi after DTMUV infection.**

**Additional file 6: DEG involved in DTMUV invasion DEF.**

**Additional file 7: DEG involved in host immune response to DTMUV in DEF.**

## Data Availability

The read sequences were deposited in the NCBI Sequence Read Archive (SRA) under accession number SRS6277135.

## Abbreviations

DTMUV: duck Tembusu virus
hpi: hour post-infection
DEFs: duck embryo fibroblasts
DEGs: differentially expressed genes
MOI: multiplicity of infection
FPKM: per kilobase of unigene per million mapped reads
GO: Gene Ontology
KEGG: Kyoto Encyclopedia of Genes and Genomes
CPE: cytopathic effects
CC: cellular component
MF: molecular functions
BP: biological process
FGF: fibroblast growth factor
TNF-α: tumor necrosis factor alpha
