# Supplementary material for: RNA-Seq analysis of duck embryo fibroblast cells gene expression during duck Tembusu virus infection
Source: Vet Res. 2022 May 18;53:34. doi: 10.1186/s13567-022-01051-y (PMC9116716; doi:10.1186/s13567-022-01051-y)
Supplement: Supplementary file 5 — Additional file 5: Information of differentially expressed genes at 60 hpi after DTMUV infection. [file 13567_2022_1051_MOESM5_ESM.docx]

DEGs involved in DTMUV invasion DEFs

|  | | | |
| --- | --- | --- | --- |
| Biological process | Gene symbol | Gene description | Source of DEGs |
| Cell cycle | GADD45B | growth arrest and DNA damage inducible beta | 12 hpi vs 24 hpi |
|  | BUB1 | BUB1 mitotic checkpoint serine/threonine kinase | 24 hpi vs 36 hpi |
|  | CCNE2 | cyclin E2 |  |
|  | CDK6 | cyclin dependent kinase 6 |  |
|  | E2F2 | E2F transcription factor 2 |  |
|  | E2F5 | E2F transcription factor 5 |  |
|  | GADD45B | growth arrest and DNA damage inducible beta |  |
|  | GADD45G | growth arrest and DNA damage inducible gamma |  |
|  | MYC | MYC proto-oncogene, bHLH transcription factor |  |
|  | SFN | stratifin |  |
|  | SKP2 | S-phase kinase associated protein 2 |  |
|  | ZBTB17 | zinc finger and BTB domain containing 17 |  |
|  | GADD45B | growth arrest and DNA damage inducible beta | 36 hpi vs 48 hpi |
|  | GADD45G | growth arrest and DNA damage inducible gamma |  |
|  | CDC20 | cell division cycle 20 | 48 hpi vs 60 hpi |
|  | GADD45B | growth arrest and DNA damage inducible beta |  |
|  | ORC1 | origin recognition complex subunit 1 |  |
|  | PLK1 | polo like kinase 1 |  |
| Focal adhesion | COL6A6 | collagen type VI alpha 6 chain | 12 hpi vs 24 hpi |
|  | IGF1 | insulin like growth factor 1 |  |
|  | PTK2 | protein tyrosine kinase 2 |  |
|  | BCAR1 | BCAR1, Cas family scaffold protein | 24 hpi vs 36 hpi |
|  | CAV2 | caveolin 2 |  |
|  | CAV3 | caveolin 3 |  |
|  | CHAD | chondroadherin |  |
|  | IGF1 | insulin like growth factor 1 |  |
|  | ITGA8 | integrin subunit alpha 8 |  |
|  | ITGB6 | integrin subunit beta 6 |  |
|  | LAMA3 | laminin subunit alpha 3 |  |
|  | MYLK4 | myosin light chain kinase family member 4 |  |
|  | PDGFRA | platelet derived growth factor receptor alpha |  |
|  | PTK2 | protein tyrosine kinase 2 |  |
|  | PXN | paxillin |  |
|  | SHC3 | SHC adaptor protein 3 |  |
|  | SPP1 | secreted phosphoprotein 1 |  |
|  | THBS4 | thrombospondin 4 |  |
|  | VEGFC | vascular endothelial growth factor C |  |
|  | COL6A1 | collagen type VI alpha 1 chain | 36 hpi vs 48 hpi |
|  | COL6A2 | collagen type VI alpha 2 chain |  |
|  | COL6A6 | collagen type VI alpha 6 chain |  |
|  | KDR | kinase insert domain receptor |  |
|  | LAMA3 | laminin subunit alpha 3 |  |
|  | LAMA5 | laminin subunit alpha 5 |  |
|  | LAMC2 | laminin subunit gamma 2 |  |
|  | LAMC3 | laminin subunit gamma 3 |  |
|  | PGF | placental growth factor |  |
|  | RELN | reelin |  |
|  | COL4A3 | collagen type IV alpha 3 chain | 48 hpi vs 60 hpi |
|  | COL6A6 | collagen type VI alpha 6 chain |  |
|  | EGF | epidermal growth factor |  |
|  | ITGB4 | integrin subunit beta 4 |  |
|  | KDR | kinase insert domain receptor |  |
|  | LAMA4 | laminin subunit alpha 4 |  |
|  | LAMC2 | laminin subunit gamma 2 |  |
|  | PTK2 | protein tyrosine kinase 2 |  |
|  | RELN | reelin |  |
|  | THBS4 | thrombospondin 4 |  |
|  | TNR | tenascin R |  |
|  | VWF | von Willebrand factor |  |
| Tight junction | GATA4 | GATA binding protein 4 | 12 hpi vs 24 hpi |
|  | RUNX1 | runt related transcription factor 1 |  |
|  | AMOTL2 | angiomotin like 2 | 24 hpi vs 36 hpi |
|  | CGN | cingulin |  |
|  | DLG2 | discs large MAGUK scaffold protein 2 |  |
|  | GATA4 | GATA binding protein 4 |  |
|  | JAM3 | junctional adhesion molecule 3 |  |
|  | MAP3K5 | mitogen-activated protein kinase kinase kinase 5 |  |
|  | PARD6A | par-6 family cell polarity regulator alpha |  |
|  | PARD6B | par-6 family cell polarity regulator beta |  |
|  | RUNX1 | runt related transcription factor 1 |  |
|  | BVES | blood vessel epicardial substance | 36 hpi vs 48 hpi |
|  | MAP3K5 | mitogen-activated protein kinase kinase kinase 5 |  |
|  | CGN | cingulin | 48 hpi vs 60 hpi |
|  | CLDN19 | claudin 19 |  |
|  | SLC9A3R1 | SLC9A3 regulator 1 |  |
| Regulation of action cytoskeleton | FGF4 | fibroblast growth factor 4 | 12 hpi vs 24 hpi |
|  | FGF5 | fibroblast growth factor 5 |  |
|  | IQGAP2 | IQ motif containing GTPase activating protein 2 |  |
|  | LPAR5 | lysophosphatidic acid receptor 5 |  |
|  | PTK2 | protein tyrosine kinase 2 |  |
|  | ARHGEF4 | Rho guanine nucleotide exchange factor 4 | 24 hpi vs 36 hpi |
|  | BCAR1 | BCAR1, Cas family scaffold protein |  |
|  | BDKRB1 | bradykinin receptor B1 |  |
|  | BDKRB2 | bradykinin receptor B2 |  |
|  | CHRM4 | cholinergic receptor muscarinic 4 |  |
|  | FGF1 | fibroblast growth factor 1 |  |
|  | FGF10 | fibroblast growth factor 10 |  |
|  | FGF19 | fibroblast growth factor 19 |  |
|  | FGF2 | fibroblast growth factor 2 |  |
|  | FGF4 | fibroblast growth factor 4 |  |
|  | FGF5 | fibroblast growth factor 5 |  |
|  | FGFR4 | fibroblast growth factor receptor 4 |  |
|  | IQGAP2 | IQ motif containing GTPase activating protein 2 |  |
|  | ITGB2 | integrin subunit beta 2 |  |
|  | ITGB6 | integrin subunit beta 6 |  |
|  | MYLK4 | myosin light chain kinase family member 4 |  |
|  | PDGFRA | platelet derived growth factor receptor alpha |  |
|  | PTK2 | protein tyrosine kinase 2 |  |
|  | PXN | paxillin |  |
|  | BDKRB1 | bradykinin receptor B1 | 36 hpi vs 48 hpi |
|  | FGF1 | fibroblast growth factor 1 |  |
|  | FGF16 | fibroblast growth factor 16 |  |
|  | FGF19 | fibroblast growth factor 19 |  |
|  | FGF3 | fibroblast growth factor 3 |  |
|  | FGF4 | fibroblast growth factor 4 |  |
|  | FGF5 | fibroblast growth factor 5 |  |
|  | FGF9 | fibroblast growth factor 9 |  |
|  | ITGAE | integrin subunit alpha E |  |
|  | LPAR5 | lysophosphatidic acid receptor 5 |  |
|  | CXCL12 | C-X-C motif chemokine ligand 12 | 48 hpi vs 60 hpi |
|  | EGF | epidermal growth factor |  |
|  | FGF1 | fibroblast growth factor 1 |  |
|  | FGF19 | fibroblast growth factor 19 |  |
|  | FGF3 | fibroblast growth factor 3 |  |
|  | FGF4 | fibroblast growth factor 4 |  |
|  | FGF5 | fibroblast growth factor 5 |  |
|  | ITGB4 | integrin subunit beta 4 |  |
|  | PTK2 | protein tyrosine kinase 2 |  |
