# Supplementary material for: RNA-Seq analysis of duck embryo fibroblast cells gene expression during duck Tembusu virus infection
Source: Vet Res. 2022 May 18;53:34. doi: 10.1186/s13567-022-01051-y (PMC9116716; doi:10.1186/s13567-022-01051-y)
Supplement: Supplementary file 6 — Additional file 6: DEG involved in DTMUV invasion DEF. [file 13567_2022_1051_MOESM6_ESM.docx]

DEGs involved in host immune response to DTMUV in DEFs

| Biological process | Gene symbol | Gene description | Source of DEGs |
| --- | --- | --- | --- |
| Cytokine-Cytokine receptor interaction | CCL20 | C-C motif chemokine ligand 20 | 12 hpi vs 24 hpi |
|  | IL10RA | interleukin 10 receptor subunit alpha |  |
|  | IL12B | interleukin 12B |  |
|  | IL12RB1 | interleukin 12 receptor subunit beta 1 |  |
|  | IL12RB2 | interleukin 12 receptor subunit beta 2 |  |
|  | IL16 | interleukin 16 |  |
|  | IL1R2 | interleukin 1 receptor type 2 |  |
|  | IL23R | interleukin 23 receptor |  |
|  | IL6 | interleukin 6 |  |
|  | IL7R | interleukin 7 receptor |  |
|  | INHBB | inhibin subunit beta B |  |
|  | TNFRSF18 | TNF receptor superfamily member 18 |  |
|  | TNFRSF4 | TNF receptor superfamily member 4 |  |
|  | TNFRSF9 | TNF receptor superfamily member 9 |  |
|  | TNFSF10 | TNF superfamily member 10 |  |
|  | TNFSF15 | TNF superfamily member 15 |  |
|  | TNFSF4 | TNF superfamily member 4 |  |
|  | TNFSF8 | TNF superfamily member 8 |  |
|  | ACKR4 | atypical chemokine receptor 4 | 24 hpi vs 36 hpi |
|  | ACVRL1 | activin A receptor like type 1 |  |
|  | BMP6 | bone morphogenetic protein 6 |  |
|  | CX3CL1 | C-X3-C motif chemokine ligand 1 |  |
|  | EDAR | ectodysplasin A receptor |  |
|  | GDF7 | growth differentiation factor 7 |  |
|  | IFNG | interferon gamma |  |
|  | IL10 | interleukin 10 |  |
|  | IL12B | interleukin 12B |  |
|  | IL12RB1 | interleukin 12 receptor subunit beta 1 |  |
|  | IL12RB2 | interleukin 12 receptor subunit beta 2 |  |
|  | IL13RA1 | interleukin 13 receptor subunit alpha 1 |  |
|  | IL17C | interleukin 17C |  |
|  | IL1R2 | interleukin 1 receptor type 2 |  |
|  | IL23R | interleukin 23 receptor |  |
|  | IL2RG | interleukin 2 receptor subunit gamma |  |
|  | IL6 | interleukin 6 |  |
|  | IL8 | interleukin 8 |  |
|  | INHBA | inhibin subunit beta A |  |
|  | INHBB | inhibin subunit beta B |  |
|  | LIF | LIF, interleukin 6 family cytokine |  |
|  | NGF | nerve growth factor |  |
|  | TNFRSF11B | TNF receptor superfamily member 11b |  |
|  | TNFRSF13B | TNF receptor superfamily member 13B |  |
|  | TNFRSF19 | TNF receptor superfamily member 19 |  |
|  | TNFRSF4 | TNF receptor superfamily member 4 |  |
|  | TNFRSF6B | TNF receptor superfamily member 6b |  |
|  | TNFRSF8 | TNF receptor superfamily member 8 |  |
|  | TNFRSF9 | TNF receptor superfamily member 9 |  |
|  | TNFSF10 | TNF superfamily member 10 |  |
|  | TNFSF11 | TNF superfamily member 11 |  |
|  | TNFSF15 | TNF superfamily member 15 |  |
|  | ACKR4 | atypical chemokine receptor 4 | 36 hpi vs 48 hpi |
|  | BMP5 | bone morphogenetic protein 5 |  |
|  | IL12RB1 | interleukin 12 receptor subunit beta 1 |  |
|  | TNFRSF13B | TNF receptor superfamily member 13B |  |
|  | TNFRSF4 | TNF receptor superfamily member 4 |  |
|  | TNFRSF8 | TNF receptor superfamily member 8 |  |
|  | TNFSF10 | TNF superfamily member 10 |  |
|  | CD40 | CD40 molecule | 48 hpi vs 60 hpi |
|  | CXCL12 | C-X-C motif chemokine ligand 12 |  |
|  | IL12B | interleukin 12B |  |
|  | IL18 | interleukin 18 |  |
|  | IL18R1 | interleukin 18 receptor 1 |  |
|  | LEPR | leptin receptor |  |
|  | NGF | nerve growth factor |  |
|  | TNFSF10 | TNF superfamily member 10 |  |
|  | TNFSF13B | TNF superfamily member 13b |  |
|  | TNFSF4 | TNF superfamily member 4 |  |
| NOD-like receptor signaling pathway | IL6 | interleukin 6 | 12 hpi vs 24 hpi |
|  | P2RX7 | purinergic receptor P2X 7 |  |
|  | TMEM173 | transmembrane protein 173 |  |
|  | TNFAIP3 | TNF alpha induced protein 3 |  |
|  | ANTXRL | ANTXR like | 24 hpi vs 36 hpi |
|  | CARD9 | caspase recruitment domain family member 9 |  |
|  | IL6 | interleukin 6 |  |
|  | IL8 | interleukin 8 |  |
|  | PLCB2 | phospholipase C beta 2 |  |
|  | PSTPIP1 | proline-serine-threonine phosphatase interacting protein 1 |  |
|  | TLR4 | toll like receptor 4 |  |
|  | P2RX7 | purinergic receptor P2X 7 | 36 hpi vs 48 hpi |
|  | CARD9 | caspase recruitment domain family member 9 | 48 hpi vs 60 hpi |
|  | IL18 | interleukin 18 |  |
|  | MAPK11 | mitogen-activated protein kinase 11 |  |
|  | PLCB2 | phospholipase C beta 2 |  |
|  | TMEM173 | transmembrane protein 173 |  |
| Toll-like receptor signaling pathway | CD86 | CD86 molecule | 12 hpi vs 24 hpi |
|  | IL12B | interleukin 12B |  |
|  | IL6 | interleukin 6 |  |
|  | CD86 | CD86 molecule | 24 hpi vs 36 hpi |
|  | IL12B | interleukin 12B |  |
|  | IL6 | interleukin 6 |  |
|  | IL8 | interleukin 8 |  |
|  | IRF5 | interferon regulatory factor 5 |  |
|  | SPP1 | secreted phosphoprotein 1 |  |
|  | TLR4 | toll like receptor 4 |  |
|  | TLR7 | toll like receptor 7 |  |
|  | TLR7 | toll like receptor 7 |  |
|  | CD40 | CD40 molecule | 48 hpi vs 60 hpi |
|  | CD80 | CD80 molecule |  |
|  | CD86 | CD86 molecule |  |
|  | IL12B | interleukin 12B |  |
|  | MAPK11 | mitogen-activated protein kinase 11 |  |
| Phagosome | ATP6V1G3 | ATPase H+ transporting V1 subunit G3 | 12 hpi vs 24 hpi |
|  | C1R | complement C1r |  |
|  | CD36 | CD36 molecule |  |
|  | COLEC11 | collectin subfamily member 11 |  |
|  | CTSS | cathepsin S |  |
|  | TAP1 | transporter 1, ATP binding cassette subfamily B member |  |
|  | TAP2 | transporter 2, ATP binding cassette subfamily B member |  |
|  | ATP6AP1 | ATPase H+ transporting accessory protein 1 | 24 hpi vs 36 hpi |
|  | COLEC11 | collectin subfamily member 11 |  |
|  | ITGB2 | integrin subunit beta 2 |  |
|  | MARCO | macrophage receptor with collagenous structure |  |
|  | MBL | mannose-binding lectin |  |
|  | NCF1 | neutrophil cytosolic factor 1 |  |
|  | NCF2 | neutrophil cytosolic factor 2 |  |
|  | RAB7B | RAB7B, member RAS oncogene family |  |
|  | THBS4 | thrombospondin 4 |  |
|  | TLR4 | toll like receptor 4 |  |
|  | TUBB1 | tubulin beta 1 class VI |  |
|  | ATP6V0D2 | ATPase H+ transporting V0 subunit d2 | 36 hpi vs 48 hpi |
|  | ATP6V1G3 | ATPase H+ transporting V1 subunit G3 |  |
|  | CD36 | CD36 molecule |  |
|  | MARCO | macrophage receptor with collagenous structure |  |
|  | COLEC11 | collectin subfamily member 11 | 48 hpi vs 60 hpi |
|  | CTSS | cathepsin S |  |
|  | MARCO | macrophage receptor with collagenous structure |  |
|  | NCF4 | neutrophil cytosolic factor 4 |  |
|  | TAP1 | transporter 1, ATP binding cassette subfamily B member |  |
|  | THBS4 | thrombospondin 4 |  |
|  | TUBB1 | tubulin beta 1 class VI |  |
| Endocytosis | PML | promyelocytic leukemia | 12 hpi vs 24 hpi |
|  | WIPF3 | WAS/WASL interacting protein family member 3 |  |
|  | AMPH | amphiphysin | 24 hpi vs 36 hpi |
|  | ARAP3 | ArfGAP with RhoGAP domain, ankyrin repeat and PH domain 3 |  |
|  | ARFGEF1 | ADP ribosylation factor guanine nucleotide exchange factor 1 |  |
|  | CAV2 | caveolin 2 |  |
|  | CAV3 | caveolin 3 |  |
|  | CBLB | Cbl proto-oncogene B |  |
|  | CYTH4 | cytohesin 4 |  |
|  | DAB2 | DAB2, clathrin adaptor protein |  |
|  | DNAJC6 | DnaJ heat shock protein family (Hsp40) member C6 |  |
|  | FGFR4 | fibroblast growth factor receptor 4 |  |
|  | GRK7 | G protein-coupled receptor kinase 7 |  |
|  | IL2RG | interleukin 2 receptor subunit gamma |  |
|  | PARD6A | par-6 family cell polarity regulator alpha |  |
|  | PARD6B | par-6 family cell polarity regulator beta |  |
|  | PDGFRA | platelet derived growth factor receptor alpha |  |
|  | WIPF3 | WAS/WASL interacting protein family member 3 |  |
|  | EPN3 | epsin 3 | 36 hpi vs 48 hpi |
|  | GRK7 | G protein-coupled receptor kinase 7 |  |
|  | HSPA8 | heat shock protein family A (Hsp70) member 8 |  |
|  | PSD2 | pleckstrin and Sec7 domain containing 2 |  |
|  | AMPH | amphiphysin | 48 hpi vs 60 hpi |
|  | DAB2 | DAB2, clathrin adaptor protein |  |
|  | HSPA2 | heat shock protein family A (Hsp70) member 2 |  |
|  | PML | promyelocytic leukemia |  |
|  | WIPF3 | WAS/WASL interacting protein family member 3 |  |
| mTOR signaling pathway | ATP6V1G3 | ATPase H+ transporting V1 subunit G3 | 12 hpi vs 24 hpi |
|  | FZD8 | frizzled class receptor 8 |  |
|  | IGF1 | insulin like growth factor 1 |  |
|  | WNT10A | Wnt family member 10A |  |
|  | WNT7B | Wnt family member 7B |  |
|  | WNT9A | Wnt family member 9A |  |
|  | DDIT4 | DNA damage inducible transcript 4 | 24 hpi vs 36 hpi |
|  | IGF1 | insulin like growth factor 1 |  |
|  | RPS6KA2 | ribosomal protein S6 kinase A2 |  |
|  | SKP2 | S-phase kinase associated protein 2 |  |
|  | SLC7A5 | solute carrier family 7 member 5 |  |
|  | WNT10A | Wnt family member 10A |  |
|  | WNT2B | Wnt family member 2B |  |
|  | WNT4 | Wnt family member 4 |  |
|  | WNT5B | Wnt family member 5B |  |
|  | WNT6 | Wnt family member 6 |  |
|  | WNT7B | Wnt family member 7B |  |
|  | ATP6V1G3 | ATPase H+ transporting V1 subunit G3 | 36 hpi vs 48 hpi |
|  | DEPTOR | DEP domain containing MTOR interacting protein |  |
|  | WNT10A | Wnt family member 10A |  |
|  | WNT16 | Wnt family member 16 |  |
|  | WNT2 | Wnt family member 2 |  |
|  | DDIT4 | DNA damage inducible transcript 4 | 48 hpi vs 60hpi |
|  | WNT2 | Wnt family member 2 |  |
|  | WNT6 | Wnt family member 6 |  |
